# Complete Genome Sequences of four DE4 cluster phages isolated using *Gordonia terrae 3612*

**DOI:** 10.17912/micropub.biology.002339

**Published:** 2026-10-07

**Authors:** Lauren Allen, Rachel Carpenter, Sara Henige, Colette Janik, Shyla Mundundi, Sandheep Ranganathan, Jeila Wofford, Sarah Ball

**Affiliations:** 1 Department of Biomedical Engineering, The Ohio State University, Columbus, OH, United States; 2 College of Medicine, The Ohio State University, Columbus, OH, United States; 3 Department of Neuroscience, The Ohio State University, Columbus, OH, United States; 4 College of Arts and Sciences, The Ohio State University, Columbus, OH, United States; 5 Department of Molecular Genetics, The Ohio State University, Columbus, OH, United States

## Abstract

Abblin, Scioto, Natkenzie, and BigHunkinEater are phages that were isolated from soil using the host, *Gordonia terrae* 3612. They all belong to the DE4 subcluster, and are predicted to by lytic based on their gene content. Their genomes range in size from 58,932-60,002bp and they contain between 89-90 protein-coding genes, of which 34.4-41.1% were assigned a putative function.

**Figure 1. Representative plaques:** **(A)** BigHunkinEater plaques, Avg. diameter=3-4mm (n=5) **(B)** Abblin plaques, Avg. diameter=2-4mm (n=5) **(C)** Scioto plaques, Avg. diameter 6-7mm (n=5), black dots are artifacts from counting plaques with a marker **(D)** Natkenzie plaques, Avg. diameter=4-5mm (n=5) Table: Genome Characteristics of Isolated phages: The sequencing and genome information for four DE4 phages isolated using *Gordonia terrae 3612* , including read coverage and number of 150 base singe-end reads, genome length, GC content, and number of predicted open reading frames.

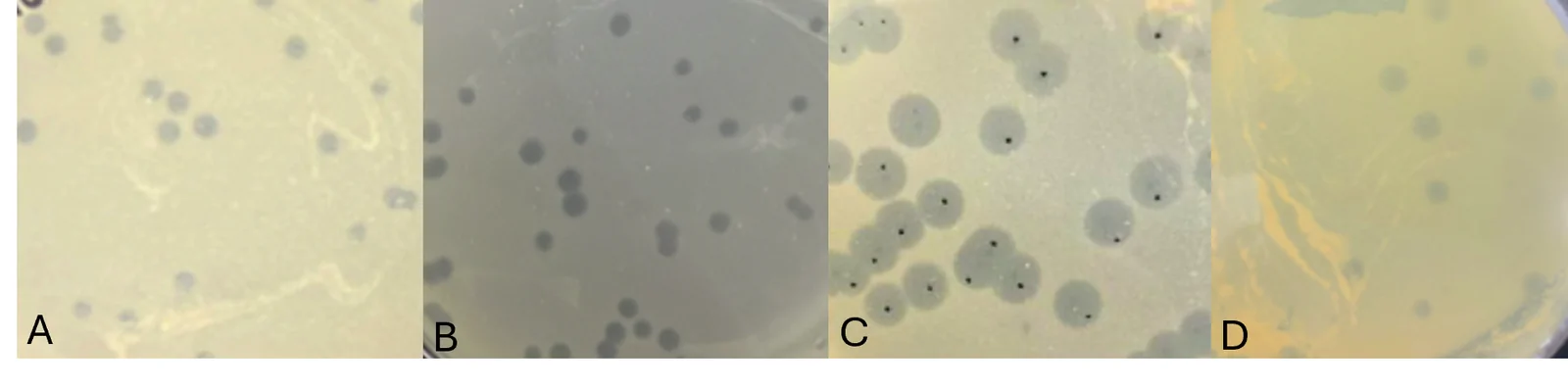



**Table:** 

Phage	GPS coordinates	Approximate read coverage	# of 150 base single-end reads	Genome length (bp)	GC content %	# of ORFs (% with predicted function)
Abblin	40.11557 N, 83.17065 W	759	318,583	60,001	67.7	89 (40.4%)
Scioto	40.079576 N, 83.112103 W	655	262,015	60,002	67.7	90 (41.1%)
Natkenzie	40.58 N, 84.33 W	632	456,081	60,001	67.7	89 (39.3%)
BigHunkinEater	39.99639 N, 83.01091 W	769	509,639	58,932	67.7	90 (34.4%)

## Description

*Gordonia terrae* and other members of the genus *Gordonia* are uncommon human pathogens but do pose a risk, primarily to immunocompromised individuals, and have been known to cause infections in the respiratory tract and soft tissues (Blanc et al., 2007; Pino-Rosa et al., 2023). Since *Gordonia terrae* has been found to be resistant to several antimicrobials, it is important to develop other possible treatments (Pino-Rosa et al., 2023). To increase our understanding of the genetic diversity of phages capable of infecting members of the genus *Gordonia* , here we present the discovery and genomic characteristics of 4 phages isolated using *G. terrae* 3612.

Bacteriophages Abblin, Scioto, Natkenzie, and BigHunkinEater were isolated via direct isolation from soil samples collected in central and eastern Ohio (GPS coordinates presented in Table 1) using standard methods described in the SEA-PHAGES guide (Poxleitner et al., 2018; Zorawik et al. 2024). The soil was suspended in PYCa media and shaken for 1 hour, centrifuged, and the supernatant then filtered through a 0.22μm filter. The filtrate was plated with top agar and incubated at 30°C for 7 days. Individual plaques were purified using three rounds of plaque streaking. All plates were incubated at 30°C for 7 days. Top agar overlay assays produced clear plaques with diameters ranging from 2-7mm (n=5) ( Figure 1 A-D).

Phage DNA was extracted from lysates using the Promega Wizard DNA Clean-Up kit and prepared for sequencing using the NEBNext Ultra II FS kit. The genomes of phages Abblin, Scioto and Natkenzie were sequenced using an Illumina MiSeq 1000 sequencer (v3 reagents) and the raw reads were assembled by Newbler v2.9 (Miller et al., 2010) with default settings and Consed v29 (Gordon and Green, 2013) was used to check accuracy, coverage, and genomic termini (Table 1). The genome of BigHunkinEater was sequenced using an Illumina NextSeq 1000 sequencer and the reads were trimmed and filtered by: cutadapt 4.7 (using the option: –nextseq-trim 30) and skewer 0.2.2 (using the options: -q 20 -Q 30 -n -l 50) prior to assembly, as stated above. (Table 1).

Based on a gene content similarity greater than 35% to phages in the Actinobacteriophage Database, all four phages were placed in the DE4 subcluster and have circularly permuted genomes (Russell & Hatfull, 2017; Pope et al. 2017). The genomes ranged in size from 58,932bp to 60,002bp and had a GC content of 67.7%.

Each genome was automatically annotated in PECAAN (Rinehart et al., 2016) using Glimmer v3.02 (Delcher et al., 2007) and GeneMarkS v4.28 (Besemer, 2005). Then, the annotation was refined using BLAST searches against the NCBI Actinobacteriophage Proteins and non-redundant protein sequences databases (Altschul et al., 1990), HHpred searches against the PDB_mmCIF70, SCOPe70, Pfam-A, NCBI_Conserved_Domains databases (Söding et al., 2005), and Phamerator using Actino_draft (Cresawn et al., 2011) and TMHMM databases (Hallgren et al., 2022) . The default setting for each software was used for the annotation. No tRNA genes were identified using Aragorn v1.2.41 (Lanslett, 2004) and tRNAscan-SE v2.012 (Lowe et al., 1997).

The genomes have 89-90 protein coding genes, with an average of 38.8% assigned a putative function (Table 1). The functions identified include both terminase small subunit and terminase large subunit, lysin A, lysin B, portal protein, and RNA ligase, which were all found in each genome.

All predicted genes were transcribed in the forward direction. Additionally, no integrase genes or other functions associated with lysogeny were identified, suggesting that these are lytic phages not capable of establishing lysogeny. Abblin, Scioto and Natkenzie are highly similar; Abblin and Natkenzie share 100 gene content similarity (GCS) whereas Scioto contains one additional gene ( *41* ), encoding a putative minor tail protein not present in other cluster DE phages but with homologs present in more distantly related phages (clusters DC and CT, to date). BigHunkinEater is more divergent from these three phages, sharing 79 % GCS.

Nucleotide sequence accession numbers

Abblin is available at GenBank Accession No. PZ492763 and Sequence Read Archive (SRA) No. SRX33845559

Scioto is available at GenBank Accession No. PZ492819 and Sequence Read Archive (SRA) No. SRX33845573

Natkenzie is available at GenBank Accession No. PZ492804 and Sequence Read Archive (SRA) No. SRX33845565

BigHunkinEater is available at GenBank Accession No. PZ492770 and Sequence Read Archive (SRA) No. SRX33845581
